# Circadian Modulation of the Antioxidant Effect of Grape Consumption: A Randomized Controlled Trial

**DOI:** 10.3390/ijerph20156502

**Published:** 2023-08-02

**Authors:** Cynthia Blanton, Biwash Ghimire, Sana Khajeh Pour, Ali Aghazadeh-Habashi

**Affiliations:** 1 Department of Nutrition and Dietetics, Idaho State University, Pocatello, ID 83209, USA; 2 Department of Biomedical and Pharmaceutical Sciences, Idaho State University, Pocatello, ID 83209, USA; biwashghimire@isu.edu (B.G.); sanakhajehpour@isu.edu (S.K.P.)

**Keywords:** circadian, grape, oxidative stress, F2-isoprostanes, tartaric acid, resveratrol, quercetin, catechin

## Abstract

Grape consumption acts on the immune system to produce antioxidant and anti-inflammatory effects. Since immune activity demonstrates circadian rhythmicity, with peak activity occurring during waking hours, the timing of grape intake may influence the magnitude of its antioxidant effect. This study followed a 2 × 2 factorial randomized, controlled design wherein healthy men and women (*n* = 32) consumed either a grape or placebo drink with a high-fat meal in the morning or evening. Urine was collected for measurements of biomarkers of oxidative stress and grape metabolites at baseline and post-meal at hour 1 and hours 1–6. F-2 isoprostane levels showed main effects of time period (baseline < hour 1 < hours 1–6, *p* < 0.0001), time (a.m. > p.m., *p* = 0.008) and treatment (placebo > grape, *p* = 0.05). Total F2-isoprostane excretion expressed as % baseline was higher in the a.m. vs. p.m. (*p* = 0.004) and in the a.m. placebo vs. all other groups (*p* < 0.05). Tartaric acid and resveratrol excretion levels were higher in the grape vs. placebo group (*p* < 0.05) but were not correlated with F-2 isoprostane levels. The findings support a protective effect of grape consumption against morning sensitivity to oxidative stress.

## 1. Introduction

Human and animal immune defense activity usually peaks during the active period in the circadian cycle [1]. The upregulation of the defense system protects against cellular damage resulting from high levels of metabolic activity and exposure to environmental challenges during waking hours. The field of chrononutrition seeks to align the circadian periodicity of physiological systems with nutrient intake to enhance human health [2,3,4,5,6].

Isoprostanes are a group of prostaglandin-like compounds produced by the free-radical-catalyzed peroxidation of arachidonic acid. They are considered reliable and specific biomarkers of oxidative stress and have been widely used in clinical and experimental studies to assess oxidative damage in various tissues and fluids. The importance of isoprostanes as oxidative stress biomarkers lies in their stability, sensitivity, and specificity. Several studies have demonstrated the utility of isoprostanes (e.g., F2-isoprostane) as biomarkers of oxidative stress in various disease states, including cardiovascular disease, cancer and neurodegenerative disorders [7,8,9,10,11].

Grape and grape-derived products are rich sources of natural compounds with potential anti-inflammatory activity. Grape polyphenols exert anti-inflammatory and antioxidant effects through interactions with molecular targets in immune system pathways [12,13]. For example, the consumption of grapes ameliorates inflammatory conditions by suppressing the production of cytokines and markers of oxidative stress [14,15,16]. These anti-inflammatory and antioxidant effects are attributed to grapes’ different components including resveratrol [17], flavonoids (catechin and quercetin) [18], anthocyanins [19], and tartaric acid [20]. These compounds demonstrated protective effects in disorders of lipid and glucose metabolism [21,22].

Immune system activity displays a circadian rhythm. Since grape components act on molecular targets regulating inflammation and oxidative defense, synchronizing grape intake to peak immune system activity might amplify the fruit’s antioxidant effects. Thus, grape consumption in the morning, when immune defenses are built, versus in the evening, when immune components are being restored/repaired, could exert more potent anti-inflammatory and antioxidant effects.

The present study used a randomized, placebo-controlled research design to test for the circadian influence of grape consumption on oxidative damage. Individuals were challenged with a high-fat meal in the morning and evening with reconstituted grape powder. High-fat meals provoke an inflammatory and oxidative stress response [23,24]. It was hypothesized that the grape treatment would more strongly suppress levels of high-fat meal-induced oxidative stress markers in the morning, when production of these molecules typically rises, compared to the evening. Further, considering the variability in polyphenol absorption across individuals due to differences in host genetics and intestinal microbiota populations [25], urine metabolites of grape polyphenols were measured. Levels of tartaric acid, resveratrol, quercetin, and catechin—urinary biomarkers of grape intake in humans [26,27,28,29,30,31]—were measured and correlated with oxidative stress biomarker levels.

## 2. Materials and Methods

### 2.1. Materials and Reagents

Resveratrol, L-(+)-tartaric acid, quercetin, (+)-catechin hydrate sulfatase (Helix pomatia, Type H-1, sulfatase ≥ 10,000 units/g), and β-glucuronidase (Helix pomatia, Type HP-2, ≥100,000 units/mL) were purchased from Sigma-Aldrich (St. Louis, MO, USA). 8-iso prostaglandin F2α and 8-iso prostaglandin F2α-d4 were purchased from Cayman Chemical Company (Ann Arbor, MI, USA). (±)-Tartaric-2,3-d2 acid was purchased from C/D/N Isotopes (QC, Pointe-Claire, QC, Canada). Fisetin and formic acid (LC-MS grade) were obtained from TCI chemicals (Portland, OR, USA). LC-MS grade water and acetonitrile were purchased from Sigma-Aldrich (St. Louis, MO, USA). A creatinine parameter assay kit was purchased from R&D systems (Minneapolis, MN, USA), and Millex-GV Filter (0.22 µm) was obtained from EMD Millipore (Burlington, MA, USA).

### 2.2. Study Design

The Idaho State University Institutional Review Board, Human Subjects Committee approved the study protocol IRB-FY2021-264. Participants provided written informed consent. Enrollment occurred between September 2021 and May 2022. The study’s clinical trials registry identification is IRB-FY2021-264 at ClinicalTrials.gov (https://clinicaltrials.gov/, accessed on 15 May 2023).

The investigation followed a 2 × 2 factorial randomized controlled design. The independent variables were time (morning vs. evening) and treatment (grape vs. placebo). The dependent variable was the urine concentration of F-2 isoprostane, a biomarker of oxidative stress. Secondary outcomes included urine levels of grape metabolites. Briefly, fasted participants attended a laboratory visit where they consumed a high-fat meal with a grape or placebo drink in the morning or evening. Urine samples were collected at baseline and 1 h post-meal in the laboratory and 1–6 h post-meal outside the laboratory. The schedule of study activities is shown in Table 1.

### 2.3. Participants

Healthy men (*n* = 13) and women (*n* = 19) between 18 and 50 years old were recruited from the local population using university electronic news platforms and paper flyers. Exclusion criteria were being employed in shift or night work; extreme early or late chronotype [32,33]; the presence of acute infection or chronic inflammatory disease; smoking; heavy aerobic exerciser; greater than moderate alcohol intake; pregnancy or lactation; use of non-steroidal anti-inflammatory medications or specified dietary supplements. Given a medium effect size and confidence level of 0.05, the goal sample size was calculated as 18–20 participants per treatment group to detect significant main effects and interactions in a 2 × 2 factorial design with 90% power.

Potential participants completed a scripted telephone screening questionnaire to assess eligibility. Those who passed the telephone screening then completed an in-person lab visit with the principal investigator that included a detailed study description, the informed consent process, and instruction on following a low-antioxidant diet and keeping food records.

Participants were instructed to follow a low-antioxidant diet and keep written food records for two days before the laboratory visit. This was included in the protocol in order to reduce the impact of the recent intake of high-antioxidant foods and beverages on the outcome measure of oxidative stress [34]. The list of foods to avoid on the low-antioxidant diet was based on the literature describing the antioxidant content of foods [35,36,37] and is shown in Supplementary Materials. The principal investigator examined the food records during the laboratory visit to confirm participant adherence to the diet during the last two days.

### 2.4. Dietary Assessment

During the laboratory visit, after the meal was completed, participants answered a diet history questionnaire to provide data on usual dietary intake. A research staff member administered to participants the National Cancer Institute’s online Diet History Questionnaire version III (DHQ III, National Institutes of Health, Applied Research Program, National Cancer Institute, Bethesda, MD, USA, 2018) [38] that examined the past 6 months with serving sizes. Output data from the DHQ III included Healthy Eating Index-2015 (HEI-2015) total and individual component scores and average daily nutrient intakes. The HEI-2015 assesses diet quality according to adherence to the US Dietary Guidelines, with scores ranging from 0 (no adherence) to 100 (optimal adherence).

Participant food records were entered into the National Cancer Institute’s online Automated Self-Administered 24-h Dietary Assessment Tool (ASA24^®®^, version 2018) [39], developed by the National Cancer Institute, Bethesda, MD, USA. Food records were analyzed by ASA24 using the Food and Nutrient Database for Dietary Studies (FNDDS) 2011–2012 and 2013–2014 [40]. Output data from the ASA24 included daily nutrient intakes.

### 2.5. Intervention

The intervention was composed of two factors: treatment and time. The treatment factor served as the antioxidant agent and was a grape drink or placebo. The time factor served as the modulator of the effect of the agent on the oxidative stress challenge (high-fat meal) and was morning or evening. The grape drink consisted of 46 g grape powder (equivalent to 1.5 servings of fresh grapes) reconstituted in 120 mL water. The grape powder was a mixture of fresh black, red and green seeded and unseeded California grapes that had been frozen, ground with food-quality dry ice, freeze-dried and reground. The control treatment was 120 mL of a placebo drink composed of a grape-like powder. The placebo powder closely matched the grape powder in sensory characteristics, dietary fiber content, sugar profile (fructose and glucose) and organic acid (tartaric, malic, and citric) profile. Artificial dyes (FD&C) were used in the placebo in place of natural colorings to avoid the addition of polyphenolic compounds from natural colorings. Flavorings used in the placebo powder contained no polyphenolic compounds or antioxidants. Both powders were prepared in 46 g packets and provided by the California Table Grape Commission (Fresno, CA, USA) for human research. The polyphenol content is listed in Table S1. Powder packets were coded so the participants and the research staff were blinded to the treatment.

High-fat meal: A single high-fat meal was used to provoke an oxidative stress response [41,42]. The meal was an egg-biscuit food composed of egg white, biscuit, cheese, and butter (Table 2). The egg yolk was avoided due to its carotenoid (antioxidant) content [43]. Egg white generally has a neutral effect on inflammatory/oxidative stress biomarker levels [44] and it is acceptable for consumption at morning and evening meals in the United States.

Test mealtimes: The laboratory visits began at 7:00 a.m. and 6:00 p.m. These times approximate the beginning of the active and rest diurnal phases, respectively, while accommodating participant availability for testing. Published data indicate higher blood and urine markers of inflammation and oxidative stress during the early active phase compared to the early rest phase [34,45,46,47,48,49]. Notably, the beginning of the active compared to the rest phase is marked by a more significant induction of a pro-inflammatory, pro-oxidant response to an inflammatory challenge [1]. Further, the principal investigator’s pilot study showed significantly higher urine malondialdehyde (a marker of oxidative stress) levels in the morning vs. evening [50].

Randomization was performed using an online research randomization generator in blocks of 8 participants. The principal investigator assigned participants to an intervention group.

### 2.6. Anthropometric Measurements, Urine Collection and Biomarker/Metabolite Analysis

Upon arrival at the laboratory visit, height was measured to the nearest 0.1 cm using a stadiometer and weight was measured to the nearest 0.1 kg using a beam scale. Body mass index was calculated as (weight in kg)/(height in meters)^2^.

A baseline urine sample was collected during the laboratory visit before test meal consumption. For the 5 h following test meal consumption, participants were instructed to drink only water and eat no food. A 1 h post-meal urine sample was collected in the laboratory before participants left for work/home, where they collected urine in a batch for the next 5 h. The urine collection schedule was based on published data demonstrating that oxidative stress biomarker levels show peak change due to spice intake within 5 h of consumption [51].

Urine samples collected outside the laboratory were stored in a refrigerator or temporarily placed in a cooler before transfer to a refrigerator. Participants delivered home-collected samples in a cooler to the investigator within 12 h of the 5 h sample collection for storage at −80 °C before analysis.

Urine was analyzed for a marker of oxidative stress, F2-isoprostane, and grape anti-inflammatory and antioxidant polyphenol metabolites (resveratrol, tartaric acid, catechin, and quercetin) using validated Liquid Chromatography-Tandem Mass Spectrometry (LC-MS/MS) methods and values were normalized to creatinine levels determined by enzyme-linked immunoassay kit. The time points for all urine analyses were baseline, 0–1 h, and 1–6 h periods. Research staff performing urine analyses were blinded to the intervention group of urine samples.

### 2.7. Sample Preparation

The frozen urine samples were thawed at room temperature and subjected to different extraction procedures and analytical methods based on the compound of interest. A brief description of these procedures is provided below.

Urinary tartaric acid was measured after slightly modifying and validating a previously published method [52]. Briefly, the urine samples were thawed and thoroughly mixed by vortexing. An aliquot of 50 µL urine sample was spiked with 100 µL of deuterated internal standard ((±)-Tartaric-2,3-d2 Acid) in 0.5% formic acid in water. The solution was diluted to 2 mL by adding 1850 µL of 0.5% formic acid, filtered through 0.22 µm filters, and injected into the LC-MS/MS system.

For the rest of the metabolites, including F2-isoprostane, resveratrol, quercetin, and catechin, previously published methods [53,54,55] were used after some modifications and validation. Briefly, an aliquot 600 µL of urine was mixed with 100 µL of 100 ng/mL fisetin and 8-iso Prostaglandin F2α-d4 as internal standards. Then, 25 µL each of beta-glucuronidase (1000 IU) and sulfatase (2.5 IU) was added to hydrolyze the glucuronide conjugates of the metabolites. Next, 25 µL of ascorbate EDTA solution containing 200 mg/mL of ascorbic acid and 1 mg/mL of EDTA was added. The mixture was incubated for 2 h at 37 °C, 500 µL cold methanol was added to quench the reaction, and then it was dried under nitrogen gas and reconstituted with 100 µL of 70% methanol. A total of 10 µL of the final solution was injected into the LC-MS/MS system.

### 2.8. LC-MS/MS Conditions

The LC-MS/MS system comprised liquid chromatography (Shimadzu, MD, USA) with a binary pump (LC-30AD), an autosampler (SIL-30AC), a controller (CBM-20A) and an ABSciex QTRAP 5500 mass spectrometer (SCIEX, Redwood City, CA, USA). The chromatograms were monitored using Analyst 1.7, and the data were analyzed using MultiQuant 3.0 software (SCIEX, Redwood City, CA, USA). The metabolites were separated using Synergi™ Fusion-RP column (2.5 µm, 100 × 2 mm) obtained from Phenomenex (Torrance, CA, USA). The mass spectrometer was operated in negative electron spray ionization (ESI) mode, and the detection was performed in multiple reaction monitoring (MRM) mode. The summary of MS conditions precursor ions (Q1), product ions (Q3), declustering potential (DP), collision energy (CE), and ESI ion modes used for analysis are listed in Table 3.

### 2.9. Statistical Analysis

Standard least squares models were used to test the fixed effects of treatment (grape and placebo), time (a.m. and p.m.), and time period (baseline, 1 h and 1–6 h period), and their interactions on urine biomarkers F2-isoprostane and grape metabolite concentrations. Biomarker values were not normally distributed; thus, they were log-transformed before analysis. Model 1 tested the main effects of treatment, time, and all three time periods (baseline, hour 1 and hours 1–6), while model 2 tested the main effects of treatment, time, and the two post-mealtime periods. When significant main effects were observed, post hoc tests were conducted using student’s *t*-test and Tukey’s multiple comparisons test. Biomarker response expressed as percent of baseline (% baseline) was examined, with baseline biomarker level included as a random independent variable. F2-isoprostane levels were significantly higher in females vs. males (*p* = 0.0004); thus, sex was included as a covariate in the analysis of F2-isoprostane data. No significant effect of sex was seen for urine grape metabolite levels. Total F2-isoprostane and grape metabolite excretion was calculated by adding hour 1 and hours 1–6 measurements. Linear regression analysis was used to test the relationship between biomarker levels and age and BMI and F2-isoprostane and metabolite levels. Dietary intakes of antioxidant micronutrients, including vitamins A, C, and E and the mineral selenium were calculated from the diet history questionnaire, which assessed baseline diet, and food records, which evaluated intake during the low-antioxidant diet periods. Micronutrient intakes during the low-antioxidant days and usual diet were compared using nonparametric Wilcoxon/Kruskal–Wallis and Chi-Square tests. Descriptive statistics are reported as mean with standard deviation and median with interquartile range. Analyses were performed using JMP Pro 16.2.0 (SAS Institute Inc., Cary, NC, USA).

## 3. Results

### 3.1. Participant Characteristics

**Figure 1:**
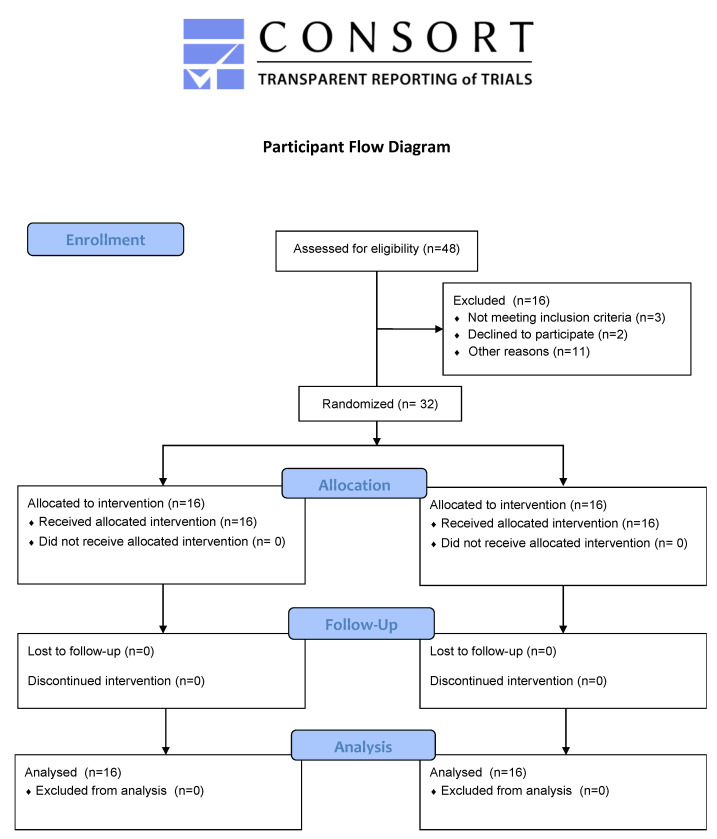
Participant flow through study protocol.

Forty-eight individuals responded to the study advertisement and were screened by telephone. Sixteen of these people did not enroll in the study due to scheduling conflicts, lack of response to invitations for an in-person screening and declining to perform study activities (Figure 1). Thirty-two participants who met the study criteria were enrolled and completed the study between December 2021 and May 2022. Participant characteristics are shown in Table 4.

### 3.2. Biomarker Response to Treatment

#### 3.2.1. Urine F2-Isoprostane Concentration

F-2 isoprostane levels (ng/mg creatinine) showed main effects of time period (baseline < 1-h < 1–6-h, *p* < 0.0001), time (a.m. > p.m., *p =* 0.008), and treatment (placebo > grape, *p =* 0.05) in the model 1 analysis. In the model 2 analysis, main effects were observed for time period (1–6-h > 1-h, *p =* 0.003) and time (a.m. > p.m., *p =* 0.001), but not treatment (*p >* 0.05) (Table 5).

**Figure 2:**
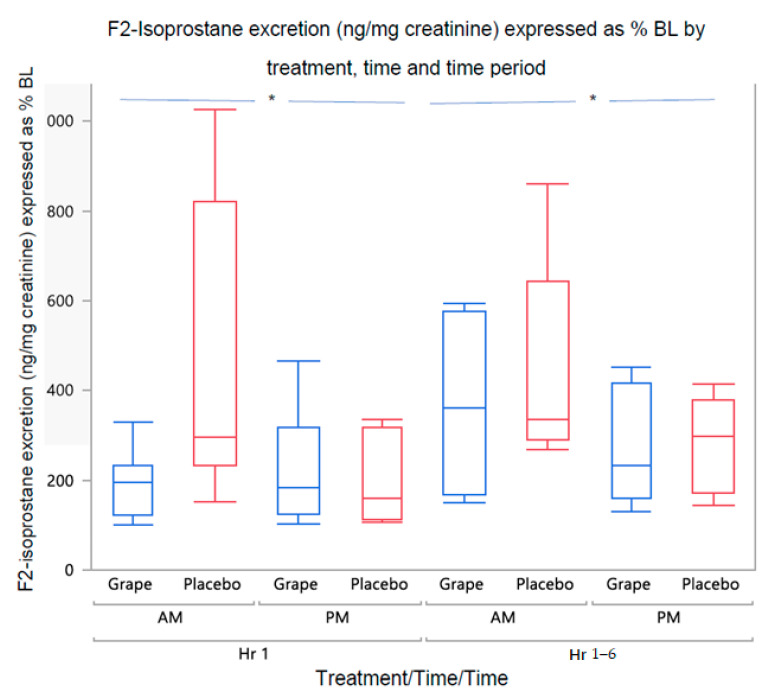
F-2 Isoprostane excretion (corrected for creatinine) expressed as % of baseline. Levels were higher in the a.m. vs. p.m. and at 1–6 vs. 1 h time periods. * Main effects of time and time period, *p* < 0.05.

Expressed as % of baseline, F2-isoprostane levels were higher in the a.m. vs. p.m. (*p* = 0.0003) and at hours 1–6 vs. hour 0–1 (*p* = 0.04) (Figure 2). A main effect was observed for treatment (grape < placebo, *p* = 0.04) when sex was excluded as a covariate in the analysis.

**Figure 3:**
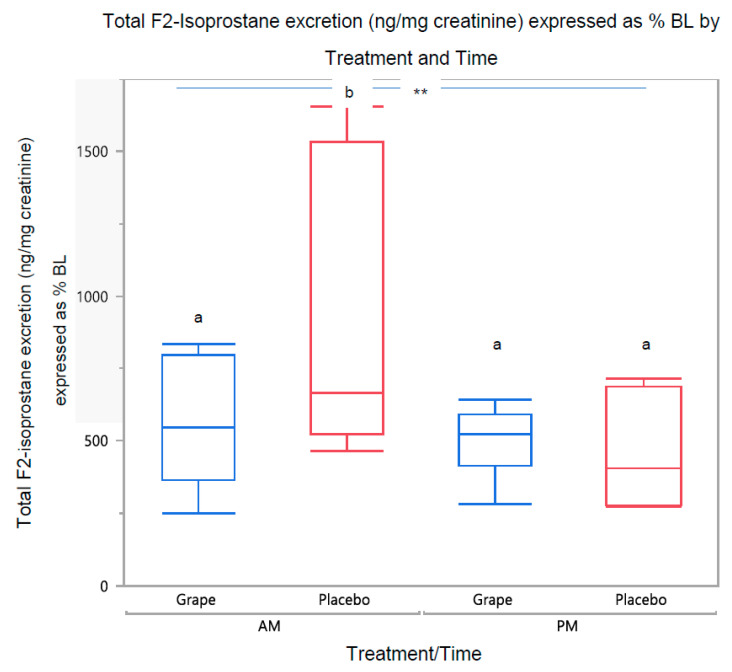
Total post-meal F2-isoprostane excretion as % of baseline. Time effect: a.m. > p.m., ** *p* = 0.0005; bars not sharing the same letter are significantly different (*p* < 0.05).

Total post-meal F2-isoprostane excretion (sum of 1 h and 1–6 h) showed a main effect of time (a.m. > p.m., *p* = 0.0007); treatment effect was not significant (*p* > 0.05). Percent of baseline total post-meal F2-isoprostane showed a main effect of time (a.m. > p.m., *p* = 0.0005) and the placebo a.m. group displayed significantly higher levels than the other groups (*p* < 0.05), (Figure 3).

F2-isoprostane levels were significantly higher in females vs. males (main effect *p* = 0.0004) and therefore sex was included as a covariate in analysis (see methods, statistical analysis). Linear regression analysis showed that F2-isoprostane excretion was unrelated to age and BMI (*p* > 0.05).

#### 3.2.2. Urine Grape Metabolite Concentrations

##### Grape Metabolite Excretion by Treatment and Time

*Tartaric acid*: Urine excretion of tartaric acid showed the main effects of treatment (grape > placebo, *p* = 0.04) and time period (1–6 h > 1 h > baseline, *p* < 0.0001) but not time (*p* > 0.05) in the model 1 analysis. The model 2 analysis also demonstrated the main effects of treatment (grape > placebo, *p* = 0.001) and time period (1–6 h > 1 h, *p* < 0.0001). Total (sum of 1 h and 1–6 h) tartaric acid showed a main treatment effect (grape > placebo, *p* = 0.007). For tartaric acid as % of baseline, main effects were seen for treatment (grape > placebo, *p* = 0.0009) and time period (1 h > 1–6 h, *p* < 0.0001) and the interaction of treatment × time period (grape 1 h > placebo 1 h > both grape and placebo 1–6 h, *p* < 0.0001).

*Catechin*: Excretion of catechin showed an effect of time period (1–6 h and 1 h > baseline, *p* < 0.0001) in the model 1 analysis. In the model 2 analysis, the impact of time period was no longer observed (no difference between 1 h and 1–6 h, *p* > 0.05). Percent of baseline catechin showed an effect of time period (1 h > 1–6 h, *p* < 0.0001). Neither time nor treatment significantly affected catechin excretion measures (*p* > 0.05).

*Quercetin*: Urine excretion of quercetin showed the main effects of time (a.m. > p.m., *p* = 0.03) and time period (1–6 h > 1 h > baseline, *p* < 0.0001) in the model 1 analysis. In the model 2 analysis, the main effects were seen for time (a.m. > p.m., *p* = 0.0001) and time period (1–6 h > 1 h, *p* = 0.003). Total quercetin showed a main effect of time (a.m. > p.m., *p* = 0.0006). The percentage of baseline quercetin showed a significant impact of the time period (1 h > 1–6 h, *p* < 0.0001) but not time (*p* > 0.05). Treatment did not demonstrate a prominent effect on measures of quercetin excretion (*p* > 0.05).

*Resveratrol*: Urine resveratrol levels showed the main effects of treatment (grape > placebo, *p* = 0.002) and time period (1 h > baseline, *p* = 0.006) but not time (*p* > 0.05) in the model 1 analysis. In the model 2 analysis, the effects of treatment and time period were no longer seen (*p* > 0.05). Neither treatment nor time affected total resveratrol excretion (*p* > 0.05). The percentage of baseline resveratrol showed a main effect of timepoint (1 h > 1–6 h, *p* < 0.0001).

##### Relationship of F2-Isoprostane to Grape Metabolites

Overall, without respect to treatment or time variables, urine total F2-isoprostane levels were inversely related to urine total tartaric acid (R^2^ = 0.17, *p* = 0.04) and directly related to total catechin (R^2^ = 0.37, *p* = 0.0003), quercetin (R^2^ = 0.74, *p* < 0.0001), and resveratrol levels (R^2^ = 0.13, *p* = 0.04).

**Figure 4:**
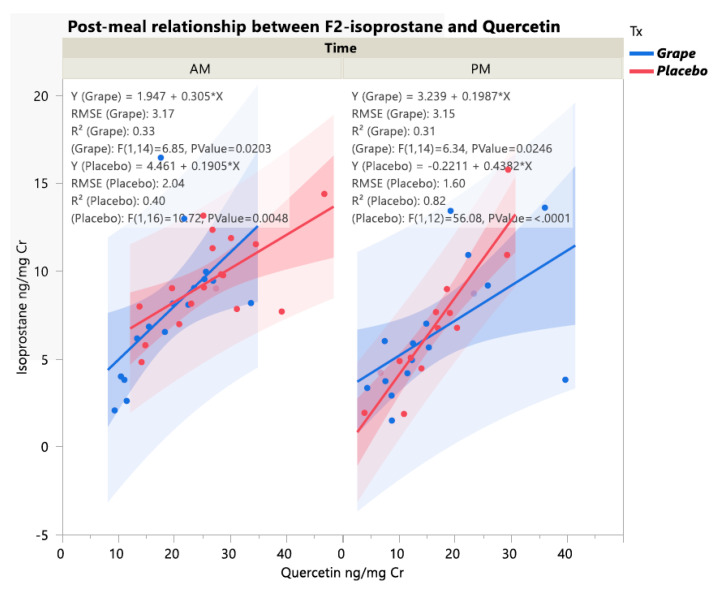
Relationship between urine post-meal F2-isoprostane and quercetin levels for each treatment and time group.

Linear regression analysis indicated that the impact of each treatment (grape and placebo) and time (a.m. and p.m.) group for the post-mealtime periods on F2-isoprostane levels were not related to urine tartaric acid levels (*p* > 0.05). Post-meal F2-isoprostane was significantly directly associated with catechin and resveratrol levels only in the placebo p.m. group (R^2^ = 0.44, *p* = 0.009 and R^2^ = 0.32, *p* = 0.04, respectively). Quercetin concentrations showed a consistent and significant direct relationship to F2-isoprostane levels across treatment/time groups, with the strongest correlation observed in the placebo p.m. group (R^2^ = 0.82, *p* < 0.0001), (Figure 4).

### 3.3. Dietary Assessment

The usual dietary intake of the participants is shown in Table 6. Participants’ HEI-2015 total scores (64.77 ± 11.79 (mean ± standard deviation)) were higher than the U.S. average for the 18–64-year-old age group (HEI = 58) [56].

Participant adherence to the low-antioxidant diet (the two days preceding each laboratory visit) was confirmed by examining food records, which showed the avoidance of high-antioxidant foods and beverages and selection of the recommended low-antioxidant items. Intakes of select antioxidant micronutrients during the low-antioxidant diet periods are shown in Table 7. Compared to their usual diet, the participants consumed significantly smaller amounts of vitamins A, C, and E during the low-antioxidant period, *p* < 0.001. Selenium intake was not significantly different between the usual diet and the low-antioxidant diet period, *p* > 0.05. Intakes of vitamins C and E were below the recommended dietary allowance values during the low-antioxidant periods, but not in the usual diet. The analysis of antioxidant micronutrient intakes per 1000 kJ demonstrated similar results, Table S2.

Antioxidant micronutrient intakes during the low-antioxidant diet and usual diet did not differ between grape and placebo, *p* > 0.05, and a.m. vs. p.m., *p* > 0.05, groups. When added as variables to the main analysis of the effects of treatment and time on F2-isoprostane excretion, antioxidant micronutrient intakes had no effect on total F2-isoprostane levels (*p* > 0.05).

## 4. Discussion

The findings of this study support the hypothesis that the timing of grape consumption modulates the body’s response to an oxidative stress challenge. Higher F2-isoprostane urine excretion following consumption of a high-fat meal was seen in the morning compared to the evening, and this response was blunted by co-ingestion of the equivalent of 1.5 servings of whole grapes. Our hypothesis is based on the circadian rhythm of immune defense [1] and evidence from chronotherapy showing differential effects of treatments and vaccines due to the timing of administration [57,58,59,60,61,62]. By identifying a circadian-sensitive antioxidant response to bioactive grape compounds, these findings support testing the impact of timed grape consumption on long-term disease outcomes. Major chronic diseases are proposed to result partly from oxidative stress [63,64,65]. The implications are that optimally timed grape intake over months/years could exert protection of body tissues from cumulative oxidative damage and support the prevention and management of disease. Grape derivatives exert antioxidant effects by activating the nuclear factor (erythroid-derived 2)-like 2 (Nrf-2) molecular signaling pathway to upregulate antioxidant enzyme action [66,67]. Future studies could be designed to test whether breakfast compared to dinner grape consumption induces antioxidant enzyme levels and activity to a greater degree and if long-term impacts on health are realized.

Support for pursuing the investigation of timed bioactive food consumption is provided by chronotherapy studies showing improved effectiveness of glucocorticoids in patients with rheumatoid arthritis when administered to target peak times of cytokine release and symptom severity [57,58,59,68,69]. Further, compared to non-timed therapy for anti-hypertensive medications, long-term chronotherapy reduces the risk of comorbidities such as cardiovascular and kidney disease [70,71]. Similarly, the therapeutic efficacy of timed administration of medications for diabetes and hypertension via implantable pumps has been recently reported [72].

The current study builds on the concept of chrononutrition in a new way. Chrononutrition research has focused on identifying optimal eating time periods for improving weight management and symptoms of metabolic disease [46,47,48,49]. Only one animal study is known to have reported a significant impact of circadian rhythm on the effect of resveratrol on oxidative stress. Gadacha et al. demonstrated an antioxidant effect of resveratrol in rats when administered during the active period but a pro-oxidant effect when administered during the rest period [73]. Therefore, failing to account for circadian rhythm could mask a significant, consistent antioxidant effect of grape consumption. Notably, F2-isoprostane levels in urine do not demonstrate circadian fluctuation [74]. Thus, the higher F2-isoprostane levels in the a.m. vs. p.m. shown in this study appear to be due to a heightened sensitivity to the high-fat meal in the morning. Our study design that included a time-of-day factor was critical in demonstrating the antioxidant effect of grapes.

In this study, we measured urine grape metabolites to assess the absorption of the grape’s antioxidant and anti-inflammatory components and identify possible correlations with the oxidative stress response. Contrary to our expectations, higher metabolite excretion was not related to lower F2-isoprostane levels in regression models. The current finding of a positive relationship between urine F2-isoprostane and quercetin concentrations has been reported previously [45]. The importance of the stronger relationship between F2-isoprostanes and quercetin in the placebo p.m. compared to other groups in the current study (Figure 3) is unclear.

The finding of higher F2-isoprostane levels and oxidative stress biomarkers in female vs. male individuals has been reported previously [75,76,77]. Thus, sex was included as a covariate in statistical analysis. Identifying sex- and time-specific outcomes related to grape and other polyphenol-rich foods in adequately powered studies will be necessary. Notably, the antioxidant effect demonstrated in this study was observed using a relatively modest dose of grapes (1.5 serving of whole grapes) and in healthy young adults consuming a generally healthful habitual diet (mean HEI-2015 score of 64.7). Investigations of the health benefits of bioactive food often involve patients with existing diseases, where an intervention effect is easier to detect [78]. Thus, the findings of this study may be relevant for the prevention of oxidative stress-related disease in currently healthy people.

Strengths of this study include the randomized, blinded, placebo-controlled design and consumption of the high-fat meal and treatment under observation. Further, participants followed a low-antioxidant diet for two days and fasted for 12 h before the study visit in order to minimize the effects of the background diet on outcomes. Adherence to the low-antioxidant diet was confirmed with diet records. Also, urine F2-isoprostane concentrations were quantified using LC/MS-MS, a more reliable method compared to enzyme-linked immunoassays [79]. Limitations are the small sample size, which limits the generalizability of results, and collection of urine by participants in the fasted state for a 1–6 h period outside of the laboratory while they were not under observation. However, asking participants to remain in the laboratory for seven hours would have negatively impacted recruitment. Further, compounds in the grape powder that were not measured in this study could have affected levels of urine F2-isoprostanes.

## 5. Conclusions

In conclusion, the results of this study demonstrate a morning vs. evening sensitivity to the antioxidant effect of grape consumption. Future investigations of the bioactive effects of food should account for the circadian rhythmicity of immune system activity.

## Figures and Tables

**Table 1:** Study schedule.

Activity	2 Days before the Lab Visit	12 h before the Lab Visit	Lab Visit	1–6 h Period Post-Meal
Low-antioxidant diet	x			
Food records	x			
Water-only fast		x		x
Urine sample *			x	x
Treatment administered			x	
Diet assessment			x	

* Baseline and 1 h post-meal, or all collected urine during 1–6 h post-meal period.

**Table 2:** Composition of the high-fat meal.

Ingredient	kJ	Fat (g)	Carbohydrate (g)	Protein (g)	(% Total Energy)		
					Fat	Carb	Protein
Biscuit (150 g)	2196	30	50	12			
Egg White (150 g)	347	0	3	15			
Cheddar Cheese (50 g)	845	18	1	11			
Butter (20 g)	598	17	0	0			
Total	3987	65	54	38	61%	23%	16%

**Table 3:** Summary of MS conditions for quantified analytes.

Analyte	Q1	Q3	DP	CE	CXP
Catechin	289.0	108.9	−90	−90	−5
Catechin	289.0	205.0	−90	−90	−15
Quercetin	300.9	150.8	−90	−90	−13
Quercetin	301.1	106.8	−90	−90	−13
Resveratrol	227.0	143.0	−90	−90	−13
Resveratrol	226.9	185.1	−75	−75	−13
PGF2α	353.0	193.0	−90	−90	−19
PGF2α	353.4	309.1	−50	−50	−13
Fisetin	285	135	−90	−90	−13
Fisetin	285	121	−90	−90	−13
PGF2α-d4	357.0	197.0	−90	−90	−19
PGF2α-d4	357.0	295.0	−80	−80	−13
Tartaric acid	149	87	−25	−20	−10
Tartaric acid	149	73	−25	−25	−10
Tartaric acid-d2	151	88	−25	−20	−10
Tartaric acid-d2	151	74	−25	−25	−10

**Table 4:** Participant characteristics (*n* = 32).

Characteristic	Mean (SD) or Number (%)	
	Females	Males
Sex	19 (59%)	13 (41%)
Age, y	31.42 (9.24)	30.08 (9.57)
Height, cm	164.23 (6.27)	177.41 (9.04)
Weight, kg	72.42 (18.39)	78.76 (11.39)
BMI, kg/m^2^	26.79 (6.28)	25.12 (3.93)

**Table 5:** Urine F2-isoprostane concentrations by treatment, time, and time period.

Time Period	Grape, a.m.		Grape, p.m.	
	Mean (SD) ng/mg Cr	Median (IQR) ng/mg Cr	Mean (SD) ng/mg Cr	Median (IQR) ng/mg Cr
0 h (baseline)	3.49 (2.39)	2.73 (3.93)	2.84 (1.58)	2.56 (1.41)
0–1 h	6.28 (4.62) *	5.11 (4.86)	6.15 (3.84)	5.98 (5.27)
1–6 h	9.25 (1.86) ^#^	9.27 (1.69)	7.01 (3.71)	5.32 (6.57)
	Placebo, a.m.		Placebo, p.m.	
0 h (baseline)	2.61 (1.30)	2.90 (2.46)	3.10 (1.95)	2.42 (2.26)
0–1 h	9.08 (2.58) ^##^	9.03 (4.08)	5.43 (3.27)	4.49 (5.73)
1–6 h	9.92 (2.62)	9.78.(4.12)	7.73 (3.89)	6.77 (4.10)

Model 1 analysis (includes all time periods): main effect of treatment (placebo > grape, *p* = 0.05); time (a.m. > p.m., *p =* 0.008); time period (hour 0 < 0–1 < 1–6, *p* < 0.0001). Model 2 analysis (excludes baseline): main effect of treatment (grape vs. placebo, *p >* 0.05); time (a.m. > p.m., *p* = 0.001); time period (1–6 h > 0–1 h, *p* = 0.003). SD = standard deviation; IQR = Interquartile Range. Within columns: 0 h: Grape vs. Placebo for a.m. and p.m. *p* > 0.05. 0–1 h: Grape < Placebo for a.m., * *p* = 0.047; Grape vs. Placebo for p.m., *p* > 0.05. 1–6 h: Grape vs. Placebo for a.m. and p.m. *p* > 0.05. Within rows: Grape: 0 h: a.m. vs. p.m. *p* > 0.05. 0–1 h: a.m. vs. p.m. *p* > 0.05. 1–6 h: a.m. > p.m. ^#^ *p* = 0.042. Placebo: 0 h: a.m. vs. p.m. *p* > 0.05. 0–1 h: a.m. > p.m. ^##^ *p* = 0.020. 1–6 h: a.m. vs. p.m. *p* > 0.05.

**Table 6:** Usual dietary intake data of participants (*n* = 32).

HEI-2015 Output	Mean (SD)	Median (IQR)
Total HEI-2015 score (maximum = 100)	64.77 (11.79)	63.43 (13.51)
Food Group (Component score maximum)		
Total Vegetables (5)	3.84 (1.05)	4.13 (2.15)
Greens and Beans (5)	4.10 (1.33)	5.00 (1.69)
Total Fruits (5)	3.49 (1.49)	3.83 (2.83)
Whole Fruits (5)	4.11 (1.39)	5.00 (1.69)
Whole Grains (10)	3.88 (2.77)	2.97 (3.00)
Dairy (10)	7.51 (2.33)	7.34 (4.61)
Total Protein Foods (5)	4.74 (0.59)	5.00 (0)
Seafood and Plant Proteins (5)	4.54 (1.07)	5.00 (0)
Fatty Acids (10)	4.38 (2.29)	4.37 (3.01)
Sodium (10)	3.29 (2.11)	3.24 (2.41)
Refined Grains (10)	7.69 (2.69)	9.08 (4.83)
Saturated Fats (10)	4.93 (2.69)	4.8 (3.88)
Added Sugars (10)	8.24 (2.04)	8.57 (2.28)

Data were collected, and Healthy Eating Index 2015 scores were calculated using the National Cancer Institute’s Diet History Questionnaire III assessing the past 6 months. SD = standard deviation; IQR = Interquartile Range.

**Table 7:** Antioxidant micronutrient intakes of participants during the low-antioxidant diet and usual diet.

	Low-Antioxidant Days		Usual Diet		*p* Value	RDA *, Age 19–50 Years
	Mean (SD)	Median (IQR)	Mean (SD)	Median (IQR)		
Total vitamin A activity (RAE), mcg	362.57 (280.43)	321.61 (338.89)	1243.26 (787.36)	1015.70 (651.27)	<0.001	900 (males), 700 (females)
Vitamin C, mg	32.84 (35.92)	20.09 (35.06)	98.90 (67.62)	95.50 (73.19)	<0.001	90 (males), 75 (females)
Vitamin E (alpha-tocopherol), mg	5.99 (3.89)	5.04 (4.52)	14.62 (10.96)	10.96 (7.7)	<0.001	15 (males and females)
Selenium, mcg	86.61 (47.24)	85.49 (56.24)	116.99 (88.70)	81.25 (68.75)	0.31	55 (males and females)

Low-antioxidant diet data were collected from food records kept by participants two days before the laboratory visit. Usual diet data were collected from a diet history questionnaire assessing the previous 6 months. SD = standard deviation; IQR = Interquartile Range; RAE = Retinol Activity Equivalent; * RDA = Recommended Dietary Allowances, US Dietary Reference Intakes. Nutrient comparisons are within rows and between the low-antioxidant days and the usual diet.

## Data Availability

The data presented in this article may be requested from the corresponding author. The protocol is available at ClinicalTrials.gov (https://clinicaltrials.gov/, accessed on 15 May 2023).

## Supplementary Materials

The following supporting information can be downloaded at https://www.mdpi.com/article/10.3390/ijerph20156502/s1. Figure S1: Urine tartaric acid excretion by treatment, time, and timepoint. Main effects were found for treatment (grape > placebo, * *p* = 0.04) and time period (1–6 h > 1 h > baseline, *** *p* < 0.0001) but not time (*p* > 0.05); Figure S2: Urine catechin excretion by treatment, time, and timepoint. A main effect was found for time period (1–6 h and 1 h > baseline, *** *p* < 0.0001). Neither time nor treatment significantly affected catechin excretion measures (*p* > 0.05); Figure S3: Urine quercetin excretion by treatment, time and, timepoint. Main effects were found for time (a.m. > p.m., *p* = 0.03) and time period (1–6 h > 1 h > baseline, *** *p* < 0.0001). Treatment did not demonstrate a significant effect on measures of quercetin excretion (*p* > 0.05); Figure S4: Urine resveratrol excretion by treatment, time, and timepoint. Main effects were found for treatment (grape > placebo, ** *p* = 0.002) and time period (1 h > baseline, *** *p* = 0.006) but not time (*p* > 0.05); Table S1: Polyphenol composition of freeze-dried grape powder; Table S2: Antioxidant nutrient intakes standardized to per 1000 kcals of participants during the low-antioxidant diet and usual diet.

## References

[1] Curtis A.M., Bellet M.M., Sassone-Corsi P., O’Neill L.A. (2014). Circadian Clock Proteins and Immunity. Immunity.

[2] Stenvers D., Scheer F., Schrauwen P., la Fleur S.E., Kalsbeek A. (2019). Circadian Clocks and Insulin Resistance. Nat. Rev. Endocrinol..

[3] Manoogian E., Panda S. (2016). Circadian Clock, Nutrient Quality, and Eating Pattern Tune Diurnal Rhythms in the Mitochondrial Proteome. Proc. Natl. Acad. Sci. USA.

[4] Panda S. (2016). Circadian Physiology of Metabolism. Science.

[5] Adafer R., Messaadi W., Meddahi M., Patey A., Haderbache A., Bayen S., Messaadi N. (2020). Food Timing, Circadian Rhythm and Chrononutrition: A Systematic Review of Time-Restricted Eating’s Effects on Human Health. Nutrients.

[6] Katsi V., Papakonstantinou I.P., Soulaidopoulos S., Katsiki N., Tsioufis K. (2022). Chrononutrition in Cardiometabolic Health. J. Clin. Med..

[7] Praticò D. (2010). The Neurobiology of Isoprostanes and Alzheimer’s Disease. Biochim. Biophys. Acta.

[8] Roberts L.J., Morrow J.D. (2000). Measurement of F(2)-Isoprostanes as an Index of Oxidative Stress in Vivo. Free Radic. Biol. Med..

[9] Basu S. (2008). F2-Isoprostanes in Human Health and Diseases: From Molecular Mechanisms to Clinical Implications. Antioxid. Redox Signal..

[10] Seet R.C.S., Lee C.-Y.J., Lim E.C.H., Tan J.J.H., Quek A.M.L., Chong W.-L., Looi W.-F., Huang S.-H., Wang H., Chan Y.-H. (2010). Oxidative Damage in Parkinson Disease: Measurement Using Accurate Biomarkers. Free Radic. Biol. Med..

[11] Greco A., Minghetti L., Levi G. (2000). Isoprostanes, Novel Markers of Oxidative Injury, Help Understanding the Pathogenesis of Neurodegenerative Diseases. Neurochem. Res..

[12] Miller S.A., White J.A., Chowdhury R., Gales D.N., Tameru B., Tiwari A.K., Samuel T. (2018). Effects of Consumption of Whole Grape Powder on Basal NF-ΚB Signaling and Inflammatory Cytokine Secretion in a Mouse Model of Inflammation. J. Nutr. Intermed. Metab..

[13] Chuang C.C., McIntosh M.K. (2011). Potential Mechanisms by Which Polyphenol-Rich Grapes Prevent Obesity-Mediated Inflammation and Metabolic Diseases. Annu. Rev. Nutr..

[14] Stamer D., Nizami S. (2017). Whole Grape Alleviates Inflammatory Arthritis through Inhibition of Tumor Necrosis Factor. J. Funct. Foods.

[15] Seymour E.M., Singer A.A., Bennink M.R., Parikh R.V., Kirakosyan A., Kaufman P.B., Bolling S.F. (2008). Chronic Intake of a Phytochemical-Enriched Diet Reduces Cardiac Fibrosis and Diastolic Dysfunction Caused by Prolonged Salt-Sensitive Hypertension. J. Gerontol. A Biol. Sci. Med. Sci..

[16] Zern T.L., Wood R.J., Greene C., West K.L., Liu Y., Aggarwal D., Shachter N.S., Fernandez M.L. (2005). Grape Polyphenols Exert a Cardioprotective Effect in Pre- and Postmenopausal Women by Lowering Plasma Lipids and Reducing Oxidative Stress. J. Nutr..

[17] Das S., Das D.K. (2007). Resveratrol: A Therapeutic Promise for Cardiovascular Diseases. Recent Pat. Cardiovasc. Drug Discov..

[18] Choy K.W., Murugan D., Leong X.-F., Abas R., Alias A., Mustafa M.R. (2019). Flavonoids as Natural Anti-Inflammatory Agents Targeting Nuclear Factor-Kappa B (NFκB) Signaling in Cardiovascular Diseases: A Mini Review. Front. Pharmacol..

[19] Costa E., Cosme F., Jordão A.M., Mendes-Faia A. (2014). Anthocyanin Profile and Antioxidant Activity from 24 Grape Varieties Cultivated in Two Portuguese Wine Regions. OENO One.

[20] Silva M.M., Lidon F.C. (2016). An Overview on Applications and Side Effects of Antioxidant Food Additives. Emir. J. Food Agric..

[21] Zhang H., Xu Z., Zhao H., Wang X., Pang J., Li Q., Yang Y., Ling W. (2020). Anthocyanin Supplementation Improves Anti-Oxidative and Anti-Inflammatory Capacity in a Dose-Response Manner in Subjects with Dyslipidemia. Redox Biol..

[22] Li D., Zhang Y., Liu Y., Sun R., Xia M. (2015). Purified Anthocyanin Supplementation Reduces Dyslipidemia, Enhances Antioxidant Capacity, and Prevents Insulin Resistance in Diabetic Patients. J. Nutr..

[23] Herieka M., Erridge C. (2014). High-Fat Meal Induced Postprandial Inflammation. Mol. Nutr. Food Res..

[24] Roehrs M., Conte L., da Silva D.T., Duarte T., Maurer L.H., de Carvalho J.A.M., Moresco R.N., Somacal S., Emanuelli T. (2017). Annatto Carotenoids Attenuate Oxidative Stress and Inflammatory Response after High-Calorie Meal in Healthy Subjects. Food Res. Int..

[25] Ozdal T., Sela D.A., Xiao J., Boyacioglu D., Chen F., Capanoglu E. (2016). The Reciprocal Interactions between Polyphenols and Gut Microbiota and Effects on Bioaccessibility. Nutrients.

[26] Meng X., Maliakal P., Lu H., Lee M.J., Yang C.S. (2004). Urinary and Plasma Levels of Resveratrol and Quercetin in Humans, Mice, and Rats after Ingestion of Pure Compounds and Grape Juice. J. Agric. Food Chem..

[27] Garcia-Perez I., Posma J.M., Chambers E.S., Nicholson J.K., Mathers J.C., Beckmann M., Draper J., Holmes E., Frost G. (2016). An Analytical Pipeline for Quantitative Characterization of Dietary Intake: Application To Assess Grape Intake. J. Agric. Food Chem..

[28] Ulaszewska M., Garcia-Aloy M., Vázquez-Manjarrez N., Soria-Florido M.T., Llorach R., Mattivi F., Manach C. (2020). Food Intake Biomarkers for Berries and Grapes. Genes Nutr..

[29] Lord R.S., Burdette C.K., Bralley J.A. (2005). Significance of Urinary Tartaric Acid. Clin. Chem..

[30] Donovan J.L., Bell J.R., Kasim-Karakas S., German J.B., Walzem R.L., Hansen R.J., Waterhouse A.L. (1999). Catechin Is Present as Metabolites in Human Plasma after Consumption of Red Wine. J. Nutr..

[31] Tsang C., Auger C., Mullen W., Bornet A., Rouanet J.M., Crozier A., Teissedre P.L. (2005). The Absorption, Metabolism and Excretion of Flavan-3-Ols and Procyanidins Following the Ingestion of a Grape Seed Extract by Rats. Br. J. Nutr..

[32] Fisher D., Lombardi D., Marucci-Wellman H., Roenneberg T. (2017). Chronotypes in the US—Influence of Age and Sex. PLoS ONE.

[33] Turco M., Corrias F., Chiaromanni M., Bano M., Salamanca M. (2015). The Self-Morningness/Eveningness (Self-ME): An Extremely Concise and Totally Subjective Assessment of Diurnal Preference. Chronobiol. Int..

[34] Hurtado-Barroso S., Quifer-Rada P., Rinaldi de Alvarenga J.F., Pérez-Fernández S., Tresserra-Rimbau A., Lamuela-Raventos R.M. (2018). Changing to a Low-Polyphenol Diet Alters Vascular Biomarkers in Healthy Men after Only Two Weeks. Nutrients.

[35] Chun O.K., Chung S.J., Song W.O. (2007). Estimated Dietary Flavonoid Intake and Major Food Sources of U.S. Adults. J. Nutr..

[36] Chun O.K., Floegel A., Chung S.J., Chung C.E., Song W.O., Koo S.I. (2010). Estimation of Antioxidant Intakes from Diet and Supplements in U.S. Adults. J. Nutr..

[37] Kim K., Vance T.M., Chun O.K. (2016). Estimated Intake and Major Food Sources of Flavonoids among US Adults: Changes between 1999-2002 and 2007-2010 in NHANES. Eur. J. Nutr..

[38] National Cancer Institute, Surveillance Research Branch. http://riskfactor.cancer.gov/DHQ/index.html.

[39] Automated Self-Administered 24-Hour (ASA24®®) Dietary Assessment Tool. https://epi.grants.cancer.gov/asa24/.

[40] Agricultural Research Service, Food Surveys Research Group USDA Food and Nutrient Database for Dietary Studies, 1.0 2004. https://data.nal.usda.gov/dataset/food-and-nutrient-database-dietary-studies-fndds.

[41] Miglio C., Peluso I., Raguzzini A., Villan D., Cesqui E., Catasta G., Toti E., Serafini M. (2013). Antioxidant and Inflammatory Response Following High-Fat Meal Consumption in Overweight Subjects. Eur. J. Nutr..

[42] Devaraj S., Wang-Polagruto J., Polagruto J., Keen C.L., Jialal I. (2008). High-Fat, Energy-Dense, Fast-Food-Style Breakfast Results in an Increase in Oxidative Stress in Metabolic Syndrome. Metabolism.

[43] Zaheer K. (2017). Hen Egg Carotenoids (Lutein and Zeaxanthin) and Nutritional Impacts on Human Health: A Review. CyTA—J. Food.

[44] Andersen C.J. (2015). Bioactive Egg Components and Inflammation. Nutrients.

[45] Dorjgochoo T., Gao Y.T., Chow W.H., Shu X.O., Yang G., Cai Q., Rothman N., Cai H., Li H., Deng X. (2012). Major Metabolite of F2-Isoprostane in Urine May Be a More Sensitive Biomarker of Oxidative Stress than Isoprostane Itself. Am. J. Clin. Nutr..

[46] Chaix A., Zarrinpar A., Miu P., Panda S. (2014). Time-Restricted Feeding Is a Preventative and Therapeutic Intervention against Diverse Nutritional Challenges. Cell Metab..

[47] Flanagan A., Bechtold D.A., Pot G.K., Johnston J.D. (2020). Chrono-Nutrition: From Molecular and Neuronal Mechanisms to Human Epidemiology and Timed Feeding Patterns. J. Neurochem..

[48] St-Onge M.-P., Ard J., Baskin M.L., Chiuve S.E., Johnson H.M., Kris-Etherton P., Varady K., on behalf of the American Heart Association Obesity Committee of the Council on Lifestyle and Cardiometabolic Health, Council on Cardiovascular Disease in the Young, Council on Clinical Cardiology (2017). Meal Timing and Frequency: Implications for Cardiovascular Disease Prevention: A Scientific Statement From the American Heart Association. Circulation.

[49] Mattson M.P., Allison D.B., Fontana L., Harvie M., Longo V.D., Malaisse W.J., Mosley M., Notterpek L., Ravussin E., Scheer F.A. (2014). Meal Frequency and Timing in Health and Disease. Proc. Natl. Acad. Sci. USA.

[50] Blanton C., Gordon B. (2020). Effect of Morning vs. Evening Turmeric Consumption on Urine Oxidative Stress Biomarkers in Obese, Middle-Aged Adults: A Feasibility Study. Int. J. Environ. Res. Public Health.

[51] Li Z., Henning S.M., Zhang Y., Zerlin A., Li L., Gao K., Lee R.P., Karp H., Thames G., Bowerman S. (2010). Antioxidant-Rich Spice Added to Hamburger Meat during Cooking Results in Reduced Meat, Plasma, and Urine Malondialdehyde Concentrations. Am. J. Clin. Nutr..

[52] Regueiro J., Vallverdú-Queralt A., Simal-Gándara J., Estruch R., Lamuela-Raventós R. (2013). Development of a LC-ESI-MS/MS Approach for the Rapid Quantification of Main Wine Organic Acids in Human Urine. J. Agric. Food Chem..

[53] Baba S., Osakabe N., Natsume M., Muto Y., Takizawa T., Terao J. (2001). In Vivo Comparison of the Bioavailability of (+)-Catechin, (-)-Epicatechin and Their Mixture in Orally Administered Rats. J. Nutr..

[54] Brose S.A., Thuen B.T., Golovko M.Y. (2011). LC/MS/MS Method for Analysis of E₂ Series Prostaglandins and Isoprostanes. J. Lipid Res..

[55] Wang L., Morris M.E. (2005). Liquid Chromatography-Tandem Mass Spectroscopy Assay for Quercetin and Conjugated Quercetin Metabolites in Human Plasma and Urine. J. Chromatogr. B Analyt. Technol. Biomed. Life Sci..

[56] National Center for Health Statistics, U.S. Department of Agriculture, Center for Nutrition Policy and Promotion (2016). What We Eat in America/National Health and Nutrition Examination Survey, 2015–2016. Healthy Eating Index—2015 Scores.

[57] Buttgereit F., Doering G., Schaeffler A., Witte S., Sierakowski S., Gromnica-Ihle E., Jeka S., Krueger K., Szechinski J., Alten R. (2010). Targeting Pathophysiological Rhythms: Prednisone Chronotherapy Shows Sustained Efficacy in Rheumatoid Arthritis. Ann. Rheum. Dis..

[58] Buttgereit F., Mehta D., Kirwan J., Szechinski J., Boers M., Alten R.E., Supronik J., Szombati I., Romer U., Witte S. (2013). Low-Dose Prednisone Chronotherapy for Rheumatoid Arthritis: A Randomised Clinical Trial (CAPRA-2). Ann. Rheum. Dis..

[59] Cutolo M. (2016). Glucocorticoids and Chronotherapy in Rheumatoid Arthritis. RMD Open.

[60] Bonten T.N., Snoep J.D., Assendelft W.J.J., Zwaginga J.J., Eikenboom J., Huisman M.V., Rosendaal F.R., van der Bom J.G. (2015). Time-Dependent Effects of Aspirin on Blood Pressure and Morning Platelet Reactivity: A Randomized Cross-over Trial. Hypertension.

[61] Hermida R.C., Ayala D.E., Fernández J.R., Mojón A., Smolensky M.H., Fabbian F., Portaluppi F. (2013). Administration-Time Differences in Effects of Hypertension Medications on Ambulatory Blood Pressure Regulation. Chronobiol. Int..

[62] Ince L.M., Barnoud C., Lutes L.K., Pick R., Wang C., Sinturel F., Chen C.-S., de Juan A., Weber J., Holtkamp S.J. (2023). Influence of Circadian Clocks on Adaptive Immunity and Vaccination Responses. Nat. Commun..

[63] Vassalle C., Bianchi S., Battaglia D., Landi P., Bianchi F., Carpeggiani C. (2012). Elevated Levels of Oxidative Stress as a Prognostic Predictor of Major Adverse Cardiovascular Events in Patients with Coronary Artery Disease. J. Atheroscler. Thromb..

[64] Pigazzani F., Gorni D., Dyar K.A., Pedrelli M., Kennedy G., Costantino G., Bruno A., Mackenzie I., MacDonald T.M., Tietge U.J.F. (2022). The Prognostic Value of Derivatives-Reactive Oxygen Metabolites (d-ROMs) for Cardiovascular Disease Events and Mortality: A Review. Antioxidants.

[65] Arfin S., Jha N.K., Jha S.K., Kesari K.K., Ruokolainen J., Roychoudhury S., Rathi B., Kumar D. (2021). Oxidative Stress in Cancer Cell Metabolism. Antioxidants.

[66] Ding Y., Li H., Li Y., Liu D., Zhang L., Wang T., Liu T., Ma L. (2020). Protective Effects of Grape Seed Proanthocyanidins on the Kidneys of Diabetic Rats through the Nrf2 Signalling Pathway. Evid. Based Complement. Altern. Med..

[67] Kode A., Rajendrasozhan S., Caito S., Yang S.-R., Megson I.L., Rahman I. (2008). Resveratrol Induces Glutathione Synthesis by Activation of Nrf2 and Protects against Cigarette Smoke-Mediated Oxidative Stress in Human Lung Epithelial Cells. Am. J. Physiol. Lung Cell. Mol. Physiol..

[68] Cutolo M. (2018). Circadian Rhythms and Rheumatoid Arthritis. Jt. Bone Spine.

[69] Ursini F., De Giorgi A., D’Onghia M., De Giorgio R., Fabbian F., Manfredini R. (2021). Chronobiology and Chronotherapy in Inflammatory Joint Diseases. Pharmaceutics.

[70] Bowles N.P., Thosar S.S., Herzig M.X., Shea S.A. (2018). Correction to: Chronotherapy for Hypertension. Curr. Hypertens. Rep..

[71] Smolensky M.H., Hermida R.C., Geng Y.J. (2020). Chronotherapy of Cardiac and Vascular Disease: Timing Medications to Circadian Rhythms to Optimize Treatment Effects and Outcomes. Curr. Opin. Pharmacol..

[72] Lee S.H., Wan Q., Wentworth A., Ballinger I., Ishida K., Collins J.E., Tamang S., Huang H.-W., Li C., Hess K. (2021). Implantable System for Chronotherapy. Sci. Adv..

[73] Gadacha W., Ben-Attia M., Bonnefont-Rousselot D., Aouani E., Ghanem-Boughanmi N., Touitou Y. (2009). Resveratrol Opposite Effects on Rat Tissue Lipoperoxidation: Pro-Oxidant during Day-Time and Antioxidant at Night. Redox Rep..

[74] Helmersson J., Basu S. (1999). F2-Isoprostane Excretion Rate and Diurnal Variation in Human Urine. Prostaglandins Leukot. Essent. Fat. Acids.

[75] Savage K., Gogarty L., Lea A., Deleuil S., Nolidin K., Croft K., Stough C. (2022). The Relationship between F(2)-Isoprostanes Plasma Levels and Depression Symptoms in Healthy Older Adults. Antioxidants.

[76] Wiener C., Rassier G.T., Kaster M.P., Jansen K., Pinheiro R.T., Klamt F., Magalhães P.V., Kapczinski F., Ghisleni G., da Silva R.A. (2014). Gender-Based Differences in Oxidative Stress Parameters Do Not Underlie the Differences in Mood Disorders Susceptibility between Sexes. Eur. Psychiatry.

[77] Brunelli E., Domanico F., La Russa D., Pellegrino D. (2014). Sex Differences in Oxidative Stress Biomarkers. Curr. Drug Targets.

[78] Forman H.J., Zhang H. (2021). Targeting Oxidative Stress in Disease: Promise and Limitations of Antioxidant Therapy. Nat. Rev. Drug Discov..

[79] Klawitter J., Haschke M., Shokati T., Klawitter J., Christians U. (2011). Quantification of 15-F2t-Isoprostane in Human Plasma and Urine: Results from Enzyme-Linked Immunoassay and Liquid Chromatography/Tandem Mass Spectrometry Cannot Be Compared. Rapid Commun. Mass Spectrom..

