# Supplementary material for: Circadian Modulation of the Antioxidant Effect of Grape Consumption: A Randomized Controlled Trial
Source: Int J Environ Res Public Health. 2023 Aug 2;20(15):6502. doi: 10.3390/ijerph20156502 (PMC10419126; doi:10.3390/ijerph20156502)
Supplement: Supplementary file 1 [file ijerph-20-06502-s001.zip › ijerph-2474701-supplementary.pdf]

Supplementary Materials

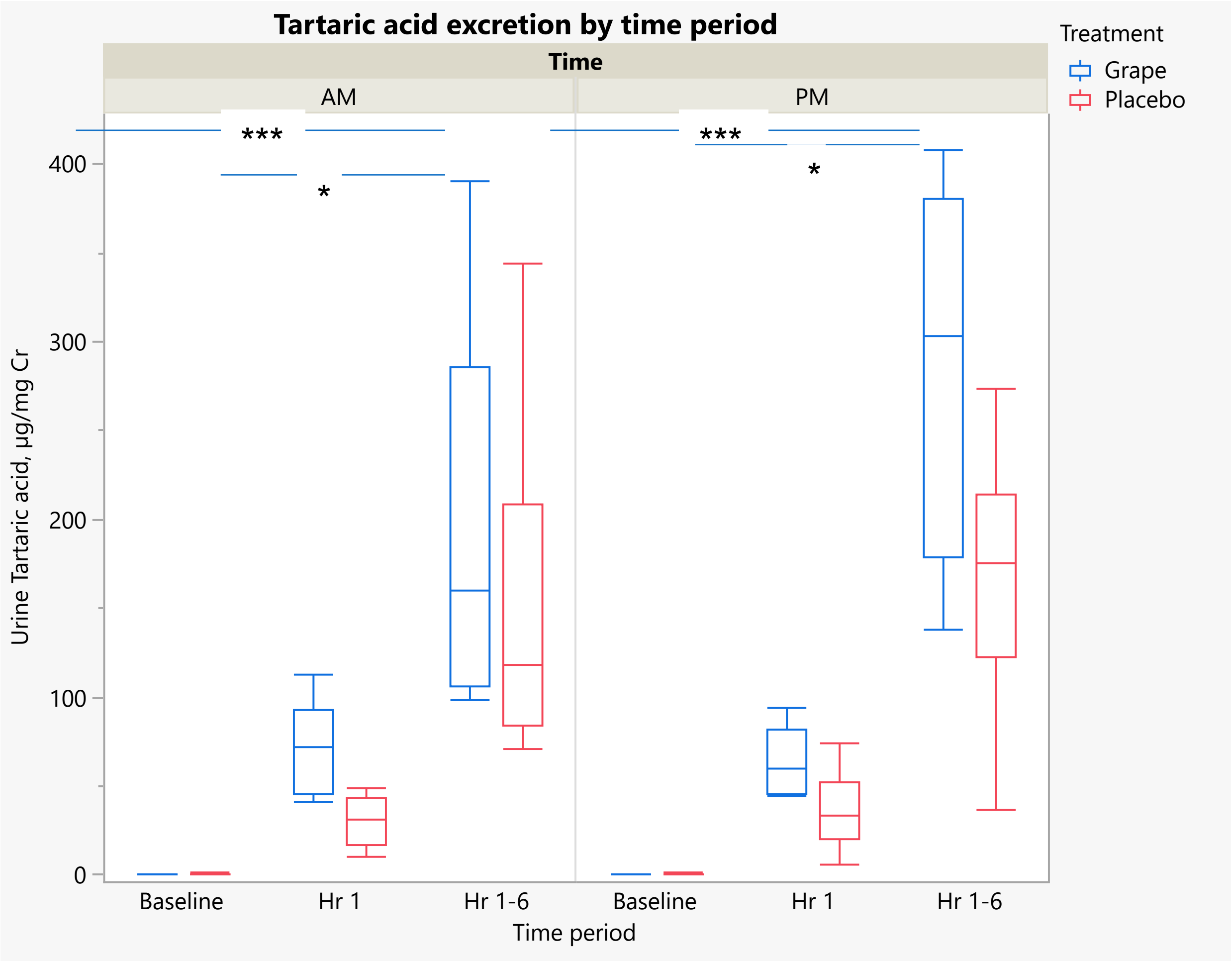

Figure S1. Urine tartaric acid excretion by treatment, time and timepoint. Main effects were found for treatment (grape > placebo, * $p = 0.04$) and time period (1–6 hours > 1 hour > baseline, *** $p < 0.0001$) but not time ($p > 0.05$).

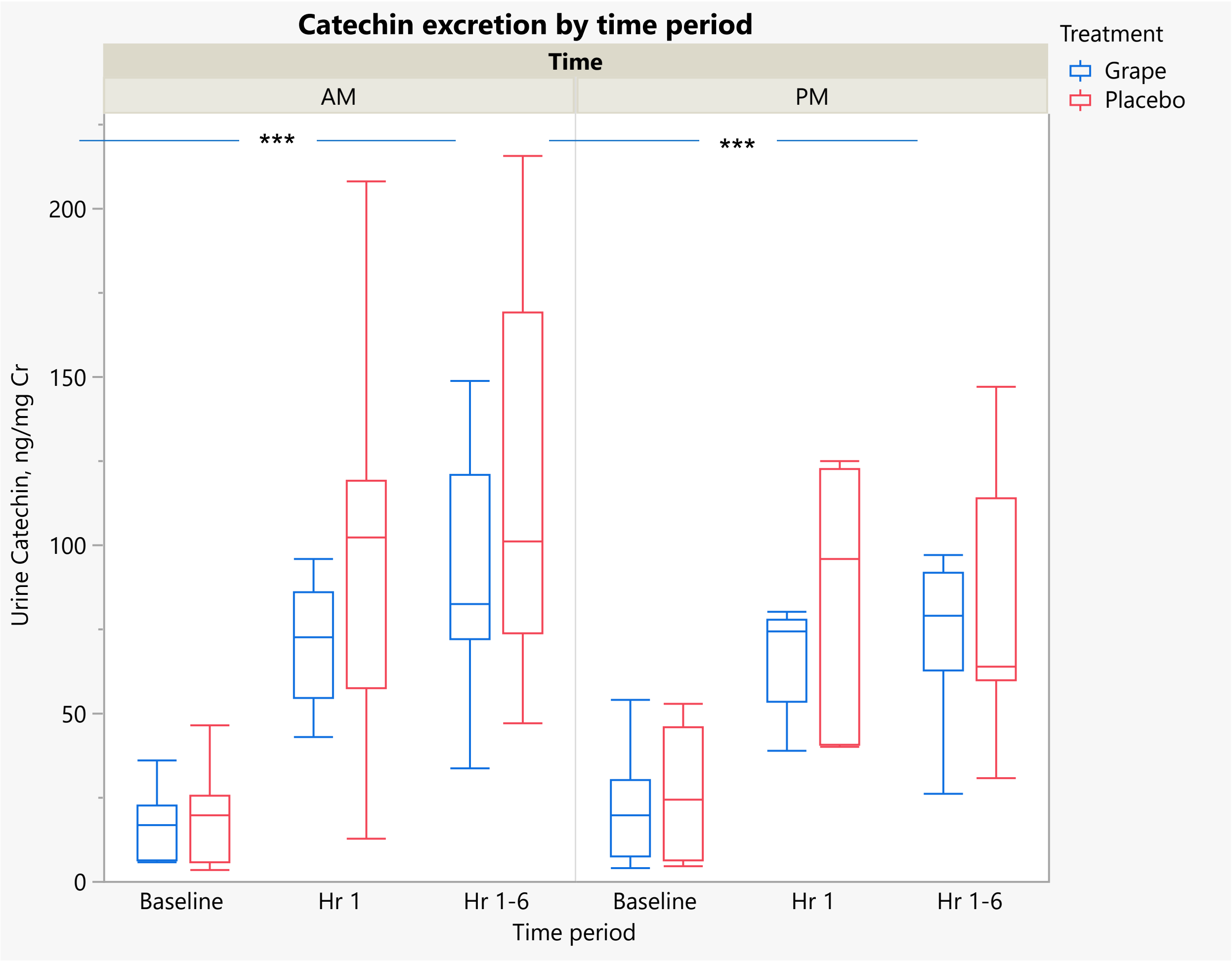

Figure S2. Urine catechin excretion by treatment, time and timepoint. A main effect was found for time period (1–6 hours and 1 hour > baseline, *** $p < 0.0001$). Neither time nor treatment significantly affected catechin excretion measures ($p > 0.05$).

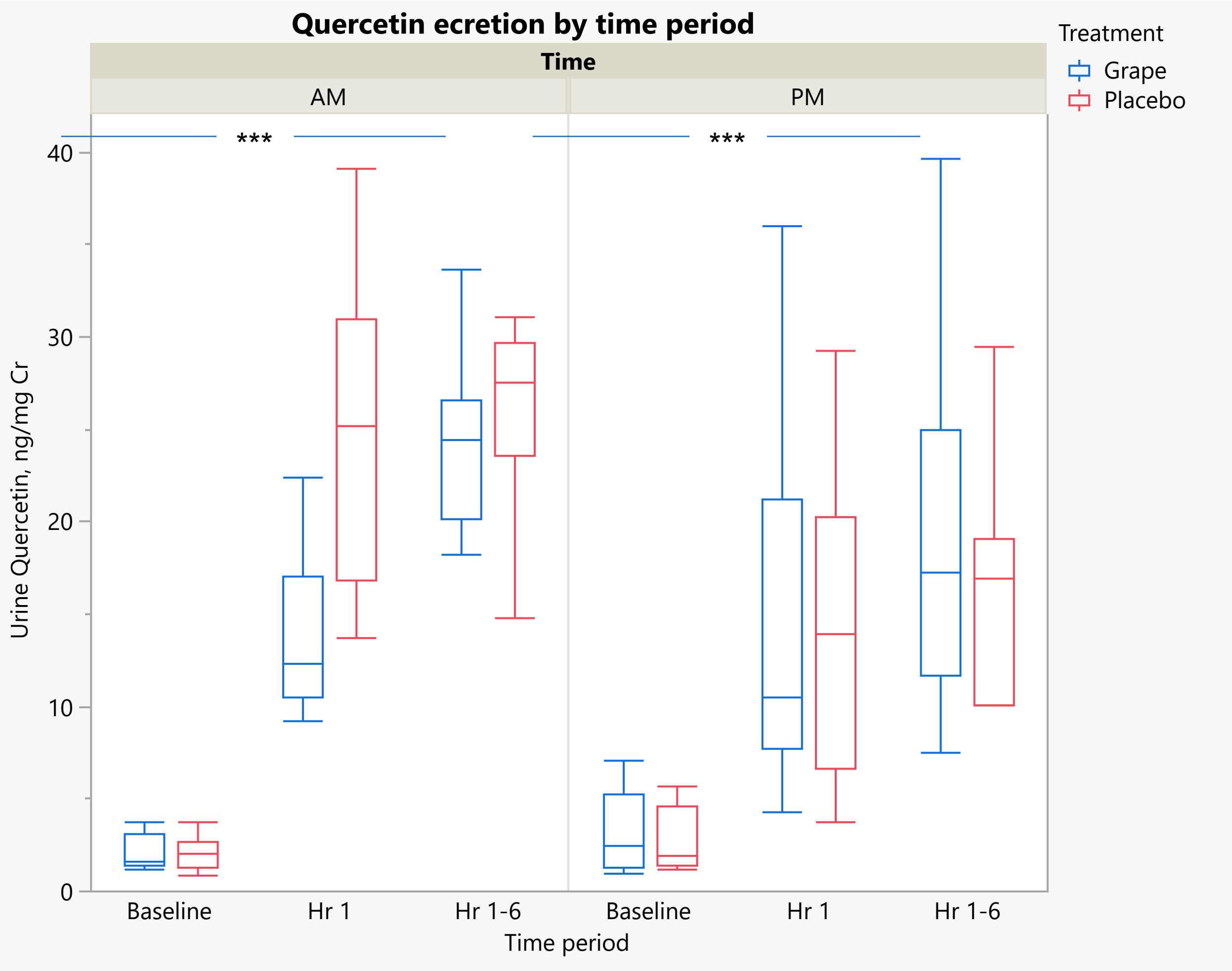

Figure S3. Urine quercetin excretion by treatment, time and timepoint. Main effects were found for time (a.m. > p.m., $p = 0.03$) and time period (1–6 hours > 1 hour > baseline, *** $p<0.0001$). Treatment did not demonstrate a significant effect on measures of quercetin excretion ($p > 0.05$).

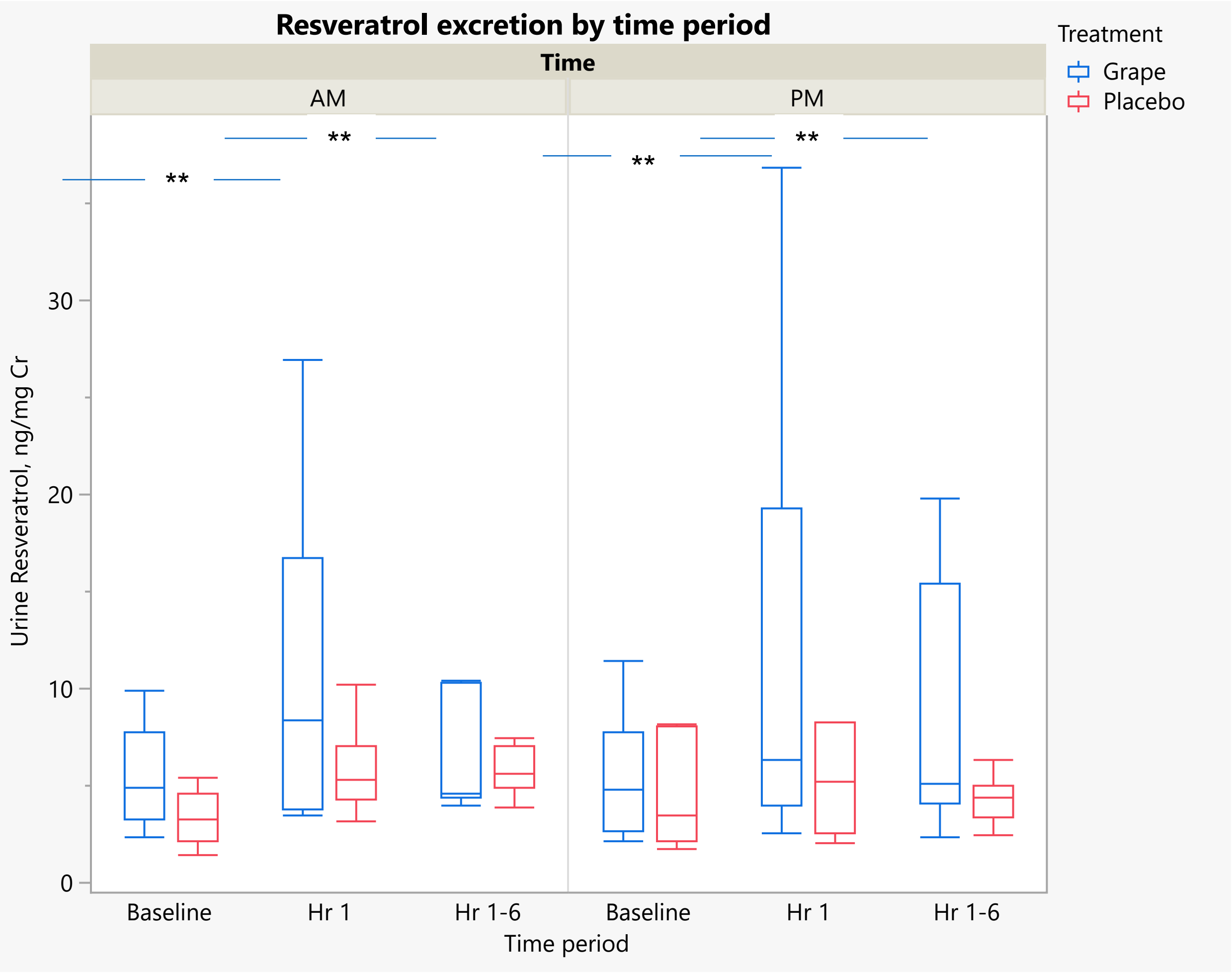

Figure S4. Urine resveratrol excretion by treatment, time and timepoint. Main effects were found for treatment (grape > placebo, ** $p = 0.002$) and time period (1 hour > baseline, *** $p = 0.006$) but not time ($p > 0.05$).

Table S1. Polyphenol composition of freeze-dried grape powder.

Polyphenols (mg/kg grape powder) ppm ± SD)	
Phenols	
Catechin	77.4 ± 12.5
Epicatechin	58.9 ± 10.2
Stilbenes	
Resveratrol	13.6 ± 1.1
Flavanols	
Quercetin	148.7 ± 10.5
Kaempferol	7.38 ± 0.62
Isorhamnetin	13.95 ± 1.84
Anthocyanins	
Cyanidin	266.7 ± 27.1
Malvidin	219.3 ± 31.3
Peonidin	47.63 ± 8.62

Table S2. Antioxidant nutrient intakes standardized to per 1000 kJ of participants during the low-antioxidant diet and usual diet.

	Low-Antioxidant Days	Usual Diet	*P* value
	Mean (SD)	Mean (SD)	
Total vitamin A activity (RAE), mcg	58.66 (38.29)	159.72 (77.33)	<0.001
Vitamin C, mg	6.04 (8.10)	12.71 (6.37)	<0.001
Vitamin E (alpha-tocopherol), mg	1.03 (0.80)	1.77 (0.63)	<0.001
Selenium, mcg	13.52 (4.45)	13.42 (2.46)	0.67

Low-antioxidant diet data were collected from food records kept by participants two days before each laboratory visit. The total number of foods recorded per participant was 8. Usual diet data were collected from a diet history questionnaire assessing the previous 6 months. SD = standard deviation; RAE = Retinol Activity Equivalent.
